# Analysis of causes and prognostic impact of tube occlusion during hyperthermic intraperitoneal chemotherapy for appendiceal pseudomyxoma peritonei

**DOI:** 10.1186/s12957-024-03412-7

**Published:** 2024-05-21

**Authors:** Qi Liu, Jie Jiao, Chengzhen Li, Yunxiang Chen, Baoxuan Wang, Jingbo Shi, Guanying Yu

**Affiliations:** 1 School of Clinical Medicine, Shandong Second Medical University, Weifang, Shandong China; 2 Department of General Surgery, Qilu Hospital of Shandong University, Jinan, China; 3 Shandong First Medical University, Jinan, China; 4 Department of Gastrointestinal Surgery, Central Hospital Affiliated to Shandong First Medical University, Jinan, China

**Keywords:** HIPEC, Tube occlusion, Gastrointestinal function, Complication

## Abstract

**Background:**

Appendiceal pseudomyxoma peritonei (PMP), a rare tumor from mucinous appendiceal origins, is treated with Cytoreductive Surgery (CRS) and Hyperthermic Intraperitoneal Chemotherapy (HIPEC). However, tubing blockages during HIPEC treatment pose a common challenge, impeding the smooth progression of therapy. Few studies to date have explored the incidence and risk factors of tube occlusion during HIPEC in patients with appendiceal PMP, as well as its adverse impact on postoperative complications.

**Methods:**

From October 2017 to June 2023, a total of 80 patients with appendiceal PMP undergoing combined CRS and HIPEC were included in this study. Tubing blockage events were strictly defined, with patients experiencing blockages during HIPEC treatment allocated to the study group, while those with unobstructed perfusion were assigned to the control group. A comparative analysis was conducted between the two groups regarding post-HIPEC health assessments and occurrence of complications. Risk factors for luminal occlusion during closed HIPEC procedures were identified through univariate and multivariate analysis of data from 303 HIPEC treatments.

**Results:**

Tubing blockages occurred in 41 patients (51.3%). The study group experienced prolonged gastrointestinal decompression time (4.1 ± 3.0 vs. 2.5 ± 1.7 days, *P* = 0.003) and prolonged time to bowel movement (6.1 ± 2.3 vs. 5.1 ± 1.8 days, *P* = 0.022) compared to the control group. There was no significant difference in the incidence of complications between the two groups. The 1-year survival rate postoperatively was 97%, and the 3-year survival rate was 81%, with no association found between tubing blockage and poorer survival. Additionally, In 303 instances of HIPEC treatment among these 80 patients, tube occlusion occurred in 89 cases (89/303, 29.4%). Multivariable logistic regression analysis revealed age, diabetes, hypertension, and pathology as independent risk factors for tube occlusion.

**Conclusion:**

Tubing blockages are a common occurrence during HIPEC treatment, leading to prolonged postoperative gastrointestinal functional recovery time. When patients are elderly and have concomitant hypertension and diabetes, along with a histological type of low-grade mucinous tumor, the risk of tube occlusion increases. However, this study did not find a significant correlation between tubing blockage and the incidence of postoperative complications or overall patient survival.

## Introduction

Pseudomyxoma peritonei (PMP) is a clinically rare condition characterized by the accumulation of gelatinous fluid and abundant mucinous masses within the abdominal cavity [1]. The most common origin is perforated appendiceal mucinous tumors [2]. Despite its rarity, the incidence of PMP has been increasing annually, estimated at approximately 1 to 3 per million per year or even higher [3]. Currently, the combined treatment modality of cytoreductive surgery (CRS) with hyperthermic intraperitoneal chemotherapy (HIPEC) has become the standard treatment for PMP since its proposition in the 1990s [4].

In 2019, the American Society of Colon and Rectal Surgeons designated CRS + HIPEC as the preferred treatment for appendiceal PMP [5]. Subsequently, in 2020, the Peritoneal Surface Oncology Group International (PSOGI) officially established international guidelines for CRS + HIPEC in the treatment of PMP [6], leading to increased attention on advanced therapies like HIPEC. Compared to CRS alone, HIPEC appears to be a more direct and effective method, allowing for thorough eradication of residual tumor tissue through localized chemotherapy perfusion [7]. However, the issue of tubing blockages during HIPEC procedures is often overlooked, despite being a prevalent challenge. Tubing blockages may arise from various causes, including postoperative fibrin congealing leading to tube obstruction, encasement and entrapment by the greater omentum, and obstruction by tumor tissue.

Tubing blockages can lead to the retention of perfusion fluid, fluctuations in intra-abdominal temperature, and decreased treatment efficacy, potentially increasing the incidence of postoperative complications. However, to date, there has been limited research exploring the relationship between tubing blockages and complications during HIPEC. This study aims to investigate not only the incidence of tubing blockages but also the associated risk factors in patients with appendiceal PMP undergoing HIPEC, as well as their adverse effects on postoperative complications. The goal is to optimize HIPEC protocols, reduce the occurrence of postoperative complications, and improve treatment outcomes and survival rates for patients.

## Materials and methods

### Patients

This study is a single-center retrospective study focusing on patients with appendiceal-origin PMP, conducted in strict accordance with the principles outlined in the Helsinki Declaration and established clinical practice guidelines. The research protocol and informed consent documentation received approval from The Ethics Committee of Central Hospital Affiliated to Shandong First Medical University (Approval No,20,240,305,004). Between October 2017 and June 2023, We included 80 patients who met the following criteria for appendiceal PMP: histologically confirmed appendiceal-origin PMP, expected survival exceeding 3 months, and complete clinical data documentation.

### Our surgical team

Our institution is a prominent center for peritoneal cancer treatment. Since 2002, under the leadership of Professor Guo, our surgical team has been engaged in laparoscopic treatment of gastrointestinal malignancies. In 2016, we introduced the HIPEC treatment technique and equipment, extending our services to a substantial number of patients with gastric cancer, colorectal cancer peritoneal metastases, and PMP. Adhering strictly to clinical guidelines, our team provides standardized CRS and HIPEC treatment protocols.

### HIPEC

For patients with appendiceal-origin PMP, CRS procedures align with standard protocols, typically involving resection of the primary lesion and right hemicolectomy [8], followed by complete cytoreduction to ensure the removal of all visible lesions [9]. Before closing the abdominal cavity, we place four catheters each in the pelvic region, splenic recess, and hepatic diaphragmatic surface. The intraperitoneal drainage tube is appropriately sized and anchored to the skin in alignment with its placement, with purse-string sutures applied when necessary, aiming to minimize tube kinking and dislocation. HIPEC treatment is administered using the BR-TRG-1 device developed by Guangzhou Baorui Medical Technology Co. LTD, with precise control of perfusion temperature (43 ± 0.5 °C), speed (600 ml/min), and duration (60 min), treatment cycles spaced 24 h apart and typically consisting of 3–4 cycles completed within 7 days. Chemotherapeutic agents utilized include loplatin, fluorouracil and raltitrexed. All drainage tubes are removed within 2 to 3 days following the completion of each treatment cycle.

During HIPEC treatment, we continuously monitor the perfusion curves of intraperitoneal inflow and outflow temperatures. Temperature sensors are placed at both the inlet and outlet, with the inlet temperature set at 43 °C. Tubing blockages can lead to a shortened effective duration of continuous perfusion treatment and may even cause fluctuations in intraperitoneal temperature. Patients experiencing tubing blockage events are categorized as the study group, while those without tubing blockage are classified as the control group.

Tubing blockage events are defined as fluctuations exceeding 0.5 °C in the perfusion curve of the outflow tube, accompanied by a decrease in outflow tube flow rate and temperature, along with a decrease in the fluid level in the reservoir bag, accompanied by abdominal distension in patients.

### Study parameters

Preoperative assessment for CRS combined with HIPEC: Clinical evaluations were performed for all patients, including the following parameters: age, gender, body mass index (BMI), history of previous abdominal surgery, history of diabetes, history of hypertension, presence of ascites, levels of CA199, CA125, CEA, organ resection status, PCI score, CC score, presence of stomas, omentectomy status, and pathological type. Postoperative observations for HIPEC: These included white blood cell count, neutrophil count, C-reactive protein levels, platelet count, temperature, hemoglobin levels, time to start enteral nutrition, time to bowel movement, length of hospital stay, and time to removal of intraperitoneal drainage tubes. Gastric decompression time, namely the time for gastric tube removal, is considered after the patient begins passing gas or when the gastric drainage volume decreases. Enteral nutrition initiation time begins after the patient has a bowel movement and attempts oral fluid diet. Complications and Follow-up: Complications occurring within 30 days after CRS + HIPEC were recorded, and severity was defined according to the Clavien-Dindo classification criteria [10]. Patients’ overall survival was monitored, with follow-up conducted via telephone every 3 to 6 months until December 2023. Furthermore, all perfusion curve records of the treatment equipment were collected for further analysis of the risk factors associated with tube occlusion.

### Statistical analysis

Categorical variables are presented as n (%) and continuous variables as mean ± standard deviation. Univariate analysis was performed using the chi-square test, Student’s t-test, or Mann-Whitney U test, multivariable analysis was conducted using a binary logistic regression model, while survival comparison was conducted using Kaplan-Meier survival analysis. All statistical analyses were carried out using SPSS 26.0 software (IBM Corporation, New York) and R software (version 4.2.1). A P-value < 0.05 was considered statistically significant.

## Results

### Comparison of patient clinical characteristics and surgical information

**Fig. 1:**
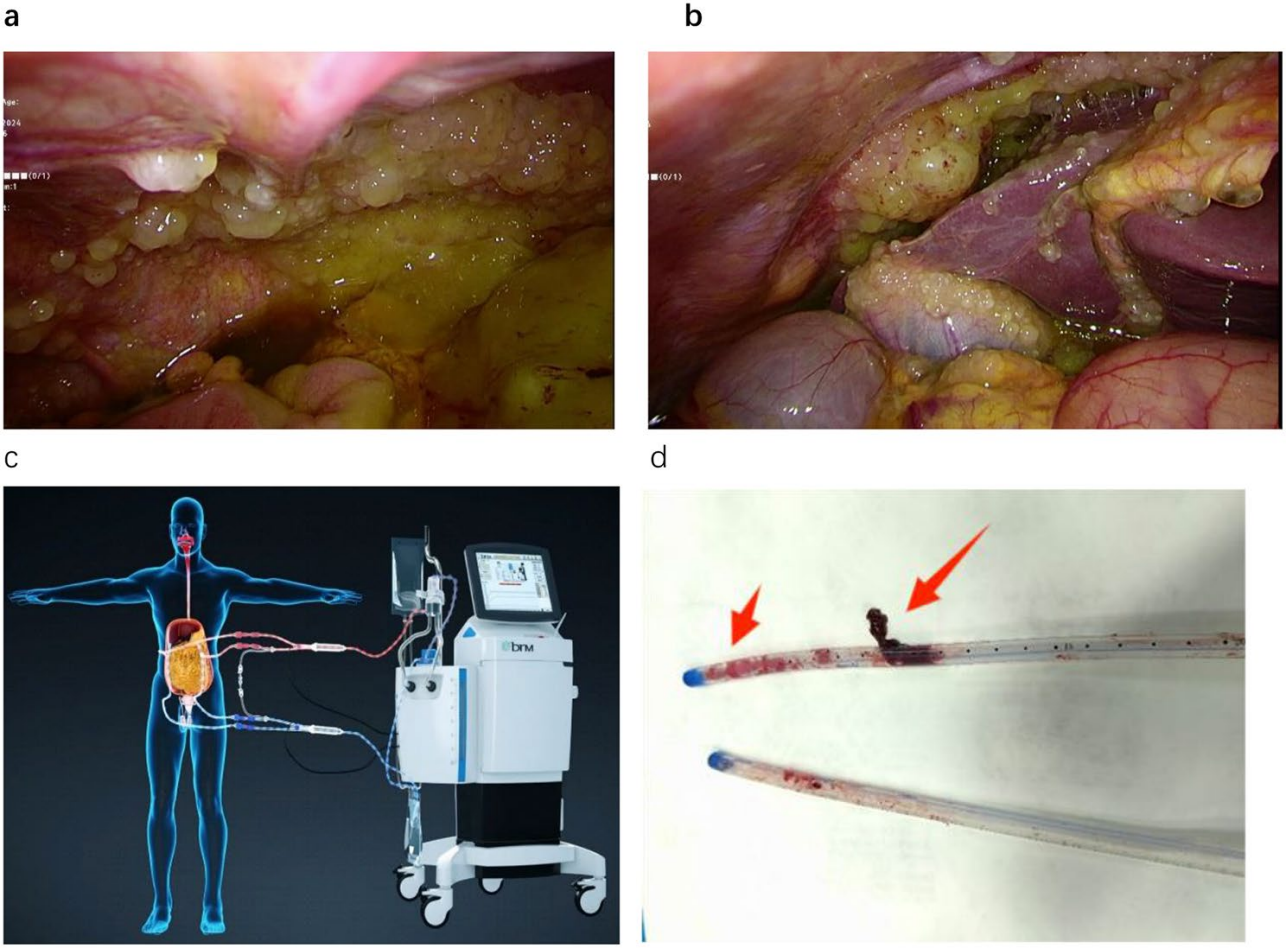
(**a** and **b**): Laparoscopic exploration. (**c**): Intraperitoneal Hyperthermic Perfusion Treatment System Schematic: Two red tubes represent inflow, while two blue tubes denote outflow. (**d**): The perfusion tubes removed from patients with tubing blockages. (Fig. 1c is cited from Guangzhou Baorui Medical Technology Co. LTD, and permission has been granted)

Between October 2017 and June 2023, a total of 80 patients with appendiceal-origin PMP were enrolled, all of whom underwent CRS combined with HIPEC (Fig. 1). The median age of these patients was 58 years, with 35 males (43.8%) and a median BMI of 23.1 kg/m2. Histologically, 42 patients (52.5%) were classified as low grade.

**Fig. 2:**
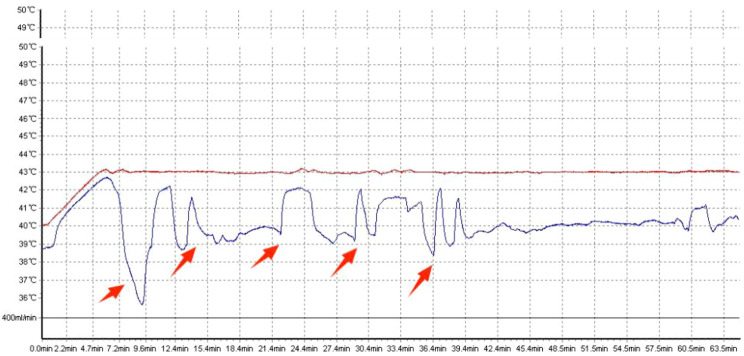
Comparison of smooth and obstructed perfusion curves. Red arrows indicate temperature fluctuations in the abdominal cavity after occlusion

During the closed-circuit HIPEC procedure, tubing blockage (Fig. 2) events occurred in 41 patients (51.3%), who were categorized into the study group, while the remaining 39 patients (48.7%) did not experience any blockage and were classified as the control group. We compared the clinical characteristics and surgical information between these two groups (Table 1). The results revealed no significant differences between the two groups in terms of gender, age, BMI, history of prior abdominal surgery, ascites, PCI score, CC score, histological type, etc. (*P* > 0.05), indicating comparability of baseline data between the two groups.

**Table 1:** Comparison of patient clinical characteristics and surgical information

Variable	Overall (*n* = 80)	Study group (*n* = 41)	Control group (*n* = 39)	*P* value
Age, year	59.9 ±10.4	61.2 ± 10.1	58.4 ±10.9	0.231
BMI, kg/m²	23.6 ±3.3	23.8± 3.7	23.4± 2.7	0.561
Gender				0.673
Male	35 (43.8%)	17	18	
Female	45 (56.2%)	24	21	
Previous Abdominal Surgery				0.252
Yes	44 (55.0%)	20	24	
No	36 (45.0%)	21	15	
Diabetes				0.064
Yes	9 (11.3%)	2	7	
No	71 (88.7%)	39	32	
Hypertension				0.035
Yes	15 (18.8%)	4	11	
No	65 (81.2%)	37	28	
Ascites				0.201
Yes	54 (67.5%)	25	29	
No	26 (32.5%)	16	10	
PCI				0.263
≥ 20	40 (50.0%)	18	22	
<20	40 (50.0%)	23	17	
CC Score				0.185
2or3	35 (43.8%)	15	20	
0or1	45 (56.2%)	26	19	
Stoma				0.665
Yes	9 (11.3%)	4	5	
No	71 (88.7%)	37	34	
Greater Omentum Resection				0.200
Yes	66 (82.5%)	36	30	
No	14 (17.5%)	5	9	
Pathology				0.268
High Grade、 Signet Ring	38 (47.5%)	17	21	
Low Grade	42 (52.5%)	24	18	

BMI: Body Mass Index. PCI: Peritoneal Cancer Index. CC: Completeness of Cytoreduction

### HIPEC Postoperative Health Assessment and Recovery Status

Among the 80 patients undergoing CRS combined with HIPEC therapy **(**Table 2**)**, 24 patients (30%) experienced postoperative fever (≥ 38 °C), and 17 patients (21.3%) had elevated BNP levels (≥ pg/mL). Additionally, the postoperative mean hemoglobin level was 110.2 ± 17.1 g/L. Inflammatory response postoperatively was evaluated using parameters such as white blood cell count, neutrophil percentage, procalcitonin, and C-reactive protein. Comparison between the study group and the control group regarding BNP, inflammatory markers, platelets, hemoglobin, and temperature showed no significant differences (*P* > 0.05).

**Table 2:** Postoperative health assessment of HIPEC patients

Variable	Overall (*n* = 80)	Study group (*n* = 41)	Control group (*n* = 39)	*P*-value
WBC,10^9^/L	7.1 ± 2.8	7.0± 2.5	7.2 ± 3.1	0.768
NEUT%	75.3 ± 13.1	75.5 ± 13.0	75.0 ± 13.3	0.889
Procalcitonin, ng/ml	0.9 ± 1.3	0.85± 1.1	1.0± 1.5	0.570
CRP, mg/L	93.5 ± 98.5	79.3 ± 59.8	109.9 ±128.7	0.194
Platelets, 10^9^/L	222.8 ± 76.0	233.4± 71.5	211.9± 79.9	0.209
Hemoglobin, g/L	110.2 ± 17.1	108.2 ± 16.0	112.3± 18.1	0.283
Body temperature, ℃	37.3 ± 0.9	37.2 ± 0.9	37.4± 0.9	0.244

WBC: White Blood Cells, CRP: C-reactive Protein, NEUT%: Neutrophil Percentage%

**Fig. 3:**
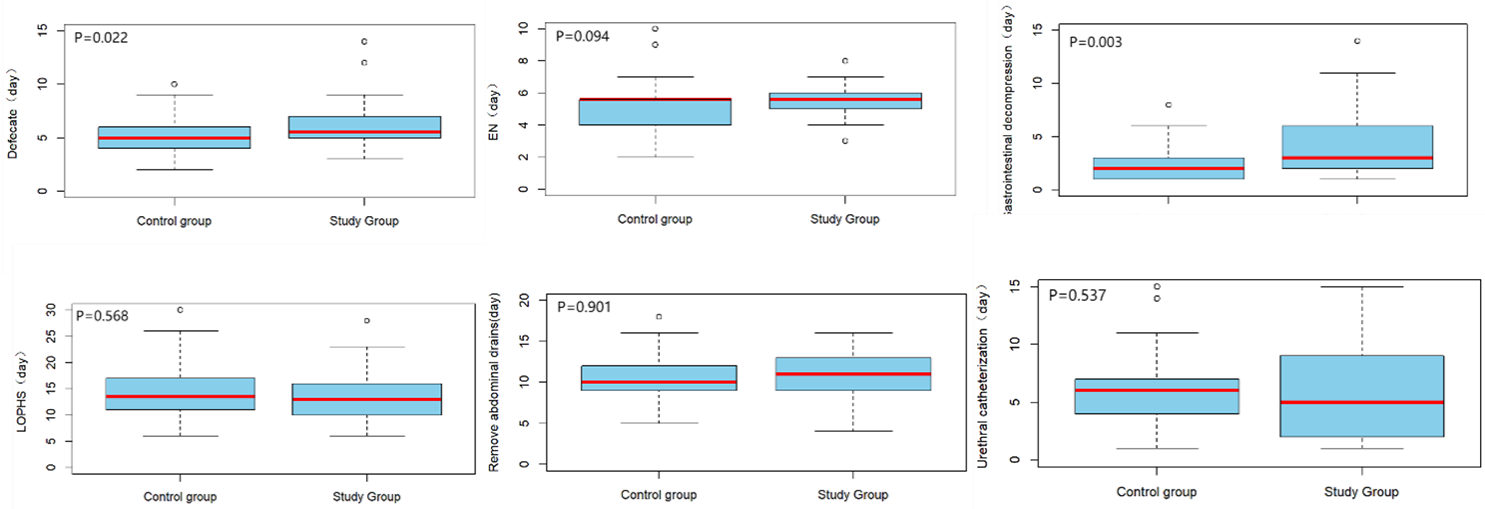
Box plots were compared, and statistical analysis was performed using Student’s t-test. EN: enteral nutrition. LOPHS: length of hospital stay

Moreover, the average length of hospital stay (LOPHS) for the 80 patients was 18.2 ± 4.9 days, with an average time to drain removal of 10.8 ± 3.1 days **(**Fig. 3**)**. Postoperative gastrointestinal function recovery was mainly assessed based on gastric decompression time, bowel movement time, and duration of parenteral nutrition. Among the 80 patients, the mean gastric decompression time was 3.3 ± 2.6 days. Compared to the control group, the study group showed significantly prolonged gastric decompression time (4.1 ± 3.0 vs. 2.5 ± 1.7 days, *P* = 0.003), and bowel movement time was also significantly prolonged (6.1 ± 2.3 vs. 5.1 ± 1.8 days, *P* = 0.022). Although there was no statistically significant difference in the duration of enteral nutrition (EN) initiation (*P* = 0.094), the study group had a slightly longer duration compared to the control group (5.9 ± 2.2 vs. 5.2 ± 1.8 days).

### Postoperative complications

Complications of grades 2 and 3 occurring within 30 days post CRS + HIPEC were documented **(**Table 3**)**. Among the 19 patients, there were a total of 28 complications, including 9 cases of intra-abdominal infection, 7 cases of surgical site infection (SSI), 2 cases of pneumonia, 3 cases of liver damage, 2 cases of neutropenia, 1 case of intra-abdominal hemorrhage, 1 case of bowel obstruction, 2 cases of anastomotic leakage, and 1 case of gastric paresis. The difference in the incidence of postoperative complications was not statistically significant (*P* = 0.561). Although tube occlusion may result in fluid retention and frequent adjustments by medical staff, its impact on incisional infection (*P* = 0.825) and intra-abdominal infection (*P* = 0.578) was not reflected in the study results.

**Table 3:** Postoperative complications following HIPEC treatment

Variable	Overall (*n* = 80)	Study group (*n* = 41)	Control group (*n* = 39)	*P*-value
All complications				0.608
Yes	19 (23.8%)	9	10	
No	61 (76.2%)	33	28	
Abdominal infection (Grade II)				0.607
Yes	9 (11.2%)	4	5	
No	71 (88.8%)	38	33	
SSI (Grade II)				0.797
Yes	7 (9.6%)	4	3	
No	73 (90.4%)	38	35	
Pneumonia (Grade IIIb)	2 (2.5%)	1	1	
Hepatic injury (Grade II)	3 (3.8%)	1	2	
Neutropenia (Grade II)	2 (2.5%)	0	2	
Intra-abdominal hemorrhage (Grade IIIb)	1 (1.3%)	0	1	
Intestinal obstruction (Grade II)	1 (1.3%)	0	1	
Anastomotic leak (Grade IIIb)	2 (2.5%)	1	1	
Gastric paresis (Grade II)	1 (1.3%)	1	0	

SSI: surgical site infection

### Survival analysis

**Fig. 4:**
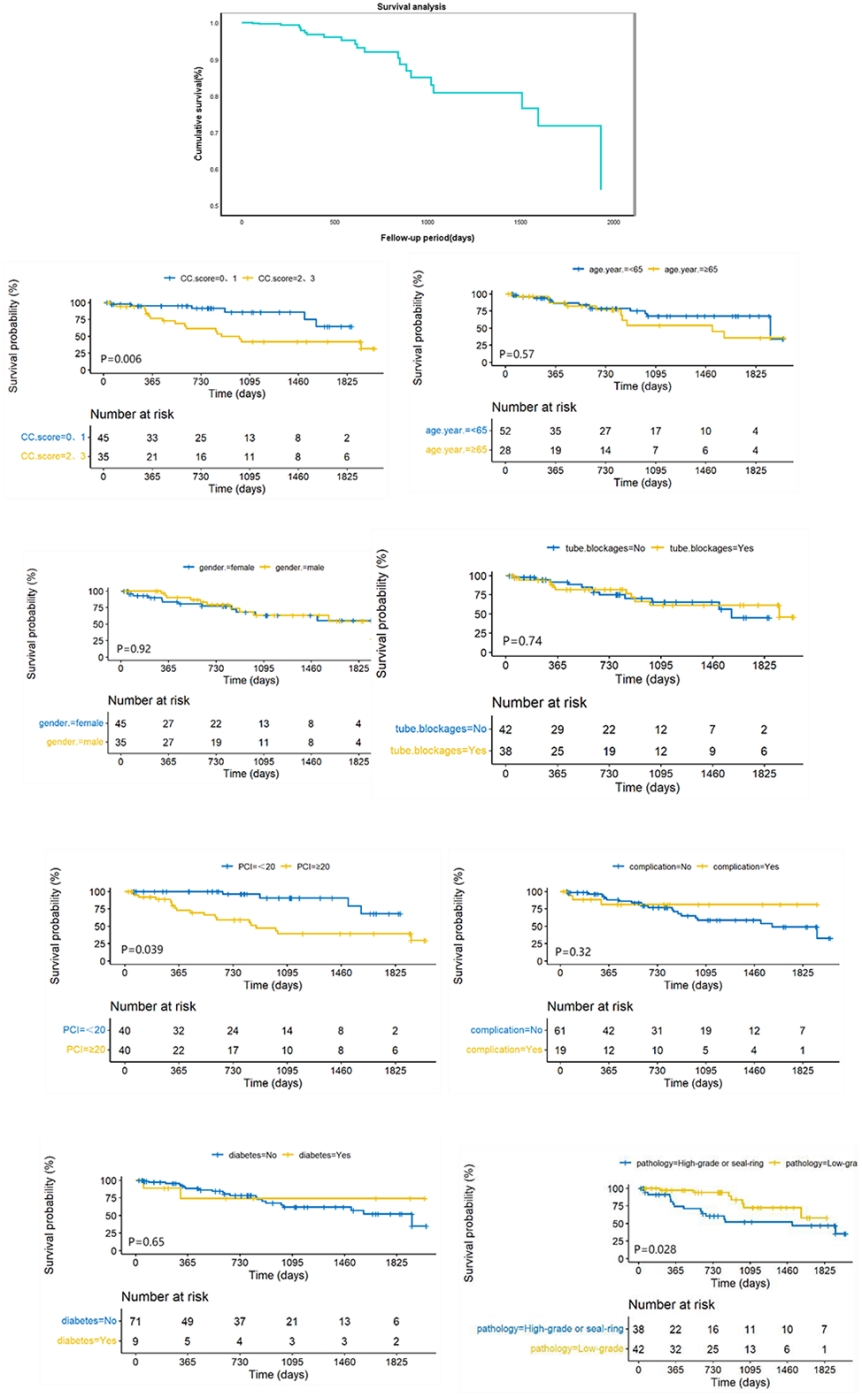
Survival analysis, performed using the log-rank test. CC: Completeness of Cytoreduction. PCI: Peritoneal Cancer Index

As of June 2023, among the 80 patients with appendiceal PMP, 23 had died **(**Fig. 4**)**. The 1-year survival rate postoperatively was 97%, and the 3-year survival rate was 81%. Kaplan-Meier log-rank test revealed no decrease in overall survival in the study group compared to the control group (*P* = 0.74). Additionally, univariate analysis showed that PCI ≥ 20 (*P* = 0.039), higher CC score (*P* = 0.006), and pathology of high grade and signet ring cell type (*P* = 0.028) were associated with adverse effects on overall survival. However, this study did not find an association between tube occlusion and poorer survival (*P* = 0.74).

### Further exploration of risk factors for tubing occlusion was conducted

A total of 303 closed HIPEC treatments were completed in 80 patients following CRS surgery, with 89 instances of tubing occlusion observed during perfusion (89/303, 29.4%) (Table 4). Univariate analysis of factors related to tubing occlusion revealed that age (*P* = 0.018), diabetes (*P* = 0.006), hypertension (*P* = 0.007), CEA (*P* = 0.02), CA199 (*P* < 0.001), organ excision (*P* = 0.013), and pathological type (*P* = 0.003) were associated with occlusion. Furthermore, binary logistic regression analysis confirmed that age (*P* = 0.01), diabetes (*P* = 0.019), hypertension (*P* = 0.017), and pathology (*P* = 0.039) were independent risk factors for occlusion.

**Table 4:** Analysis of risk factors for tube occlusion In 303 HIPEC procedures

Risk factors	comparison	Univariate analysis		Multivariate analysis	
		OR (95%CI)	*P*-value	OR (95%CI)	*P*-value
Age, year	≥ 60 VS<60	1.71 (1.03–2.83)	0.037	2.10 (1.18–3.76)	0.012
Gender	Male vs. Female	1.46 (0.88–2.42)	0.143		
BMI, kg/m²	<28 VS ≥ 28	0.55 (0.23–1.29)	0.173		
Diabetes	Yes vs. No	4.01 (1.38–11.67)	0.011	6.11 (1.35–27.70)	0.019
Hypertension	Yes vs. No	2.66 (1.28–5.51)	0.008	2.57 (1.18–5.62)	0.017
Abdomen PSH	Yes vs. No	1.01 (0.61–1.67)	0.954		
Ascites	Yes vs. No	1.51 (0.89–2.55)	0.126		
CA-125, U/mL	≤ 35 VS > 35	0.63 (0.35–1.14)	0.130		
CEA, ng/mL	≤ 5 VS>5	2.25 (1.33–3.79)	0.002	1.26 (0.68–2.34)	0.450
CA19-9, U/mL	≤ 27 VS>27	2.81 (1.64–4.81)	< 0.001	1.36 (0.69–2.65)	0.366
Visceral Resection	Yes vs. No	8.38 (1.11–63.13)	0.039	1.21 (0.70–2.85)	0.998
PCI	≤ 20 vs. >20	0.61 (0.37-1.00)	0.053		
CC Score	0、1 vs. 2、3	1.32 (1.04–1.67)	0.022	1.25 (0.67–2.34)	0.477
Stoma	Yes vs. No	1.44 (0.65–3.18)	0.358		
Omentum Resection	Yes vs. No	1.98 (0.95–4.15)	0.068		
Pathology	Low vs. High	2.34 (1.39–3.95)	0.001	2.01 (1.04–3.87)	0.037

BMI: Body Mass Index. PSH: Past Surgical History. PCI: Peritoneal Cancer Index. CC: Completeness of Cytoreduction

## Discussion

Combining CRS with HIPEC offers additional clinical benefits in patients with appendiceal PMP. Studies have shown that compared to surgery alone or systemic chemotherapy, the CRS + HIPEC regimen significantly reduces tumor recurrence rates, improves long-term survival rates, and reduces the occurrence of recurrences [8, 11, 12]. To achieve the maximum clinical benefit of HIPEC, the residual tumor diameter should be controlled to less than 0.25 centimeters postoperatively [13, 14]. The key mechanism of action of HIPEC lies in its continuous cyclic perfusion, which can mechanically clear residual cancer cells and micrometastases from the peritoneal cavity [15]. During the HIPEC treatment, fluid movement generates shear forces [16], directly leading to tumor cell death, and promotes tumor cell apoptosis through tissue flushing [17].

This study found that approximately 51.3% of patients experienced tube occlusion, indicating the severity of tube blockage issues during HIPEC and emphasizing the need for close attention to this problem. Catheter obstruction not only affects the smooth operation of continuous cyclic perfusion but may even lead to the failure of HIPEC treatment. Furthermore, fluctuation of intra-abdominal temperature between high and low temperatures may cause adverse effects on patients [18]. Direct local side effects include intestinal wall edema, intestinal perforation, intestinal fistula, anastomotic leakage, bleeding, and gastrointestinal dysfunction [19–22]. As treatment progresses, fluctuations in intra-abdominal temperature may lead to systemic changes, including heart failure, arrhythmia, bone marrow suppression, liver damage, and neurological disorders [19, 23, 24]. Hendrix et al. [25] found that in patients undergoing CRS-HIPEC treatment, the occurrence of severe hyperthermia (esophageal temperature ≥ 39.5 °C) increased the incidence of postoperative complications. Although our study did not find an increase in the occurrence of post-HIPEC treatment complications due to catheter obstruction.

This study observed that in some patients undergoing HIPEC treatment, BNP levels increased, possibly due to increased cardiac workload caused by the entry of chemotherapy drugs into the circulatory system, leading to cardiac stress response [26]. However, we did not find a correlation between catheter obstruction and elevated BNP levels. Additionally, in our study, tube blockage was found to lead to gastrointestinal dysfunction. This may be attributable to occlusion-induced fluid retention and fluctuations in intra-abdominal temperature, as well as variations in intra-abdominal pressure, which may result in the abnormal function of the intestinal vagus nerve.

In the long-term outcomes of this study, tube occlusion did not affect overall survival. Current research indicates that factors such as pathology type, preoperative PCI, CC score, and tumor markers like CA19-9 and CA-125 are crucial determinants of survival outcomes [27–29]. Individual variations exist in patient tolerance to complications such as gastrointestinal dysfunction and fluid retention caused by tube occlusion, which likely do not severely impact their overall survival. Additionally, our comprehensive treatment approach, including nutritional support and infection control, along with meticulous postoperative care and rehabilitation, helps alleviate both the short-term and long-term effects of these complications.

Due to the observed adverse effects of tube occlusion during HIPEC, we further investigated potential mechanisms and identified hypertension, age, diabetes, and pathological conditions as risk factors. Firstly, Elderly patients often display vascular aging which can lead to weakened vessel elasticity, reduced blood flow, and increased viscosity of luminal secretions like mucus [30]. Additionally, thickened secretions in the elderly, exacerbated in high-temperature environments, heighten the risk of drainage tube occlusion [31]. Secondly, diabetes and hypertension induce systemic microcirculatory disturbances, impeding the normal clearance of luminal secretions such as mucus, thereby resulting in accumulation within the drainage tube. Vascular changes cause endothelial injury and inflammation, further promoting the formation and adhesion of surrounding substances, thereby increasing the risk of drainage tube occlusion [32]. Lastly, Low-grade pseudomyxoma peritonei (PMP) is characterized by band-like or island-like tumor features, sparse cell distribution, and mild dysplasia, often exhibiting increased mucus production during cell division [33]. This high secretory activity leads to the accumulation of a large amount of mucus in the abdominal cavity, resulting in poorly flowing ascites [34]. In contrast, high-grade PMP presents as clustered tumor cells or irregular glandular structures floating in mucus. The intracellular mucus components vary and exhibit marked severe dysplasia, facilitating the aggregation of tumor cells into cancer nodules. Although high-grade PMP tumor cells continue to produce mucus and form ascites, the mucinous content [35] in the ascites is significantly lower compared to low-grade PMP. Therefore, patients with low-grade PMP have a significantly increased risk of tube occlusion, as their gelatinous or jelly-like ascites are more prone to causing obstructions [36].

Fortunately, based on the treatment experience of HIPEC over the past five years, we can share some technical details to prevent and address poor circulation caused by luminal obstruction. Initially, it is advisable to place the inlet tube in the hepatorenal recess or hepatosplenic recess for optimal therapeutic effect, while the outlet tube should be positioned at the bottom of the pelvis. Subsequently, after excluding the risk of postoperative hemorrhage, it is advisable to consider the prophylactic administration of low-molecular-weight heparin anticoagulant to decrease the viscosity of intraperitoneal fluids and prevent occlusion. Finally, prior to perfusion, water should be introduced through the outlet and the outlet should be repeatedly compressed to dislodge any tissue blockage. In case of suspected luminal obstruction, adjusting the positions of the inlet and outlet tubes and partially withdrawing the outlet tube from the abdominal cavity are viable options, albeit requiring aseptic technique. Overall, under conditions tolerable to the patient, maximizing the volume of water input and the duration of continuous flow is recommended.

Although this study provides initial insights into the impact of catheter obstruction on patients with appendiceal PMP following HIPEC, there are several limitations. Firstly, the study sample is relatively small, potentially leading to selection bias, which may affect the generalizability and representativeness of the study results. Secondly, this study was conducted at a single medical center, which may have regional and population-specific limitations. Additionally, the results of this study may only apply to patients with appendiceal PMP, and the applicability to other types of cancers remains to be further validated. Finally, occlusion may be influenced by various other factors, such as the mechanical reasons for tubing occlusion and catheter migration, which were not excluded in this study.

## Conclusion

This study demonstrates the occurrence of catheter obstruction in patients with appendiceal PMP and explores its potential impact on postoperative gastrointestinal function and survival rates. Despite certain limitations, the study provides important insights for clinical practice, aiding in a deeper understanding of the mechanisms underlying catheter obstruction and guiding the optimization of HIPEC protocols to reduce the occurrence of postoperative complications, thereby improving treatment outcomes and survival rates for patients.

## Data Availability

The datasets used and/or analysed during the current study are available from the corresponding author on reasonable request.

## Abbreviations

CRS: Cytoreductive Surgery
HIPEC: Hyperthermic Intraperitoneal Chemotherapy
CC: Completeness of Cytoreduction
PCI: Peritoneal Cancer Index
BMI: Body Mass Index
OS: Overall Survival
PSOGI: Peritoneal Surface Oncology Group International
